# A Lipidomics Atlas of Selected Sphingolipids in Multiple Mouse Nervous System Regions

**DOI:** 10.3390/ijms222111358

**Published:** 2021-10-21

**Authors:** Chunyan Wang, Juan Pablo Palavicini, Xianlin Han

**Affiliations:** 1 Barshop Institute for Longevity and Aging Studies, University of Texas Health Science Center at San Antonio, 4939 Charles Katz Drive, San Antonio, TX 78229, USA; chunyanw123@gmail.com (C.W.); PalaviciniJ@uthscsa.edu (J.P.P.); 2 Department of Medicine, Division of Diabetes, University of Texas Health Science Center at San Antonio, San Antonio, TX 78229, USA

**Keywords:** lipidomics, sphingolipid, nervous system, lipid profiling, ceramide synthases

## Abstract

Many lipids, including sphingolipids, are essential components of the nervous system. Sphingolipids play critical roles in maintaining the membrane structure and integrity and in cell signaling. We used a multi-dimensional mass spectrometry-based shotgun lipidomics platform to selectively analyze the lipid species profiles of ceramide, sphingomyelin, cerebroside, and sulfatide; these four classes of sphingolipids are found in the central nervous system (CNS) (the cerebrum, brain stem, and spinal cord) and peripheral nervous system (PNS) (the sciatic nerve) tissues of young adult wild-type mice. Our results revealed that the lipid species profiles of the four sphingolipid classes in the different nervous tissues were highly distinct. In addition, the mRNA expression of sphingolipid metabolism genes—including the ceramidase synthases that specifically acylate the N-acyl chain of ceramide species and sphingomyelinases that cleave sphingomyelins generating ceramides—were analyzed in the mouse cerebrum and spinal cord tissue in order to better understand the sphingolipid profile differences observed between these nervous tissues. We found that the distinct profiles of the determined sphingolipids were consistent with the high selectivity of ceramide synthases and provided a potential mechanism to explain region-specific CNS ceramide and sphingomyelin levels. In conclusion, we portray for the first time a lipidomics atlas of select sphingolipids in multiple nervous system regions and believe that this type of knowledge could be very useful for better understanding the role of this lipid category in the nervous system.

## 1. Introduction

As proteins, lipids are essential cellular components in cells, with more than 100,000 different lipid species predicted to make up the mammalian lipidome [1,2]. The diversity of lipids derives from the existence of numerous and complicated functional (head) groups, variable aliphatic chain lengths, the number and location of double bonds within the aliphatic chains, and the modification of hydroxyl group(s), among other variations in special chemical properties. Lipids with various structures participate not only as membrane barriers, but also play important roles in membrane integrity, cellular transport, energy storage, and signaling transduction [3,4].

The nervous system tissue is one of the richest mammalian organs in terms of lipid diversity and content [5]. Notably, lipids play critical roles in the maintenance of the normal structure and functioning of the nervous system, with myelin standing out as an essential lipid-rich component. In fact, lipid metabolism abnormalities are strongly associated with abnormal neurodevelopment and a plethora of neurological disorders [6,7,8]. Therefore, determining the profiles and distribution of lipid species in the nervous tissues is particularly important in the field of neuroscience.

Lipidomics is a rapidly expanding research frontier that studies cellular lipidomes on a large scale [9,10,11]. Sphingolipids are essential for brain development and homeostasis, and changes in their molecular species profiles are indicative of an aberrant metabolism and informative about the underlying molecular mechanisms driving such abnormalities [12,13]. For example, the myelin sphingolipid content and/or composition is critical for myelination, myelin maintenance, and myelin function [14,15]. In the current study, we focused on identifying and quantifying the molecular species of four selected sphingolipid classes—including ceramide (Cer), sphingomyelin (SM), cerebroside, and sulfatide—and on determining their distribution in the different nervous system tissues from adult mice using a technology pioneered by our group known as multi-dimensional mass-spectrometry-based shotgun lipidomics (MDMS-SL) [16,17,18,19,20]. The lipidomics data obtained from this study should not only fill the knowledge gap regarding the normal levels of different sphingolipid species in different regions of the nervous system, but should also increase our understanding of the differential synthesis of these sphingolipid species in the different nervous tissues. The sphingolipid profiling data from the different central nervous system (CNS) regions could provide us with a rough picture of the different levels of these lipid species in the different cell types, since the abundance of neurons and some major glial cells—e.g., oligodendrocytes—differs between the nervous tissues (e.g., the proportion of neurons is highest in the cerebrum, while the proportion of oligodendrocytes is highest in the spinal cord). Such information should offer useful insight into the molecular mechanisms underpinning lipid-mediated neurological disorders and diseases.

## 2. Results

In this study, the individual molecular species of the different sphingolipid classes—including Cer, SM, cerebroside, and sulfatides—present in the mouse central nervous system (CNS) (i.e., cerebrum, brain stem, spinal cord) and peripheral nervous system (PNS) (i.e., sciatic nerve) were identified and quantified using MDMS-SL. We found that the distinct molecular species profiles of the sphingolipid classes were present in the different nerve tissues, as described in detail below.

**Figure 1:**
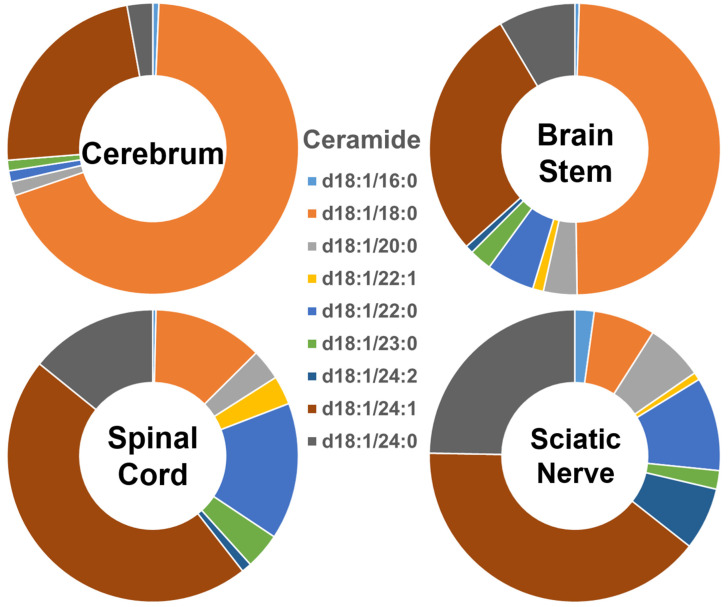
Comparison of ceramide molecular species profiles in cerebrum, brain stem, spinal cord, and sciatic nerve tissues from 2 mo-old mice. Levels of individual ceramide molecular species were assessed via shotgun lipidomics; the relative abundance of each species compared to the total class content is displayed in the form of pie graphs (Excel).

The composition of each of the ceramide species within the four different nervous system tissues is shown in Figure 1, and their absolute levels expressed as nmol/mg of the total protein content are listed in Supplementary Figure S1. In the cerebrum, Cer species containing long-chain fatty amides (i.e., those with 16 to 21 carbon atoms in the aliphatic chain) made up more than 70% of the total Cer content, whereas those containing very-long-chain fatty acids (i.e., those with an aliphatic chain longer than 22 carbons) made up less than 30% of the total Cer content. The proportion of these two different-length ceramide species present in the brain stem was around 50% each. In contrast, the group of Cer species containing individual very-long-chain fatty amides was predominant, making up over 80% of the total Cer content in the spinal cord and sciatic nerve, whereas those containing long-chain fatty amides made up less than 20% of that content. Clearly, the ceramide species containing 18:0 fatty amide (i.e., Cer (d18:1/18:0)) were predominant in the gray-matter-rich CNS regions (i.e., cerebrum). The abundance of this species gradually decreased in the brain stem, spinal cord, and sciatic nerve. In contrast to the composition of Cer (d18:1/18:0), Cer (d18:1/24:1), and Cer (d18:1/24:0) were the predominant species in the sciatic nerve and spinal cord, with their relative abundance in the different nervous system tissues being the opposite of that of Cer (d18:1/18:0). The absolute levels of each Cer species detected (expressed as nmol/mg of the total protein content) are listed in Supplementary Table S2.

**Figure 2:**
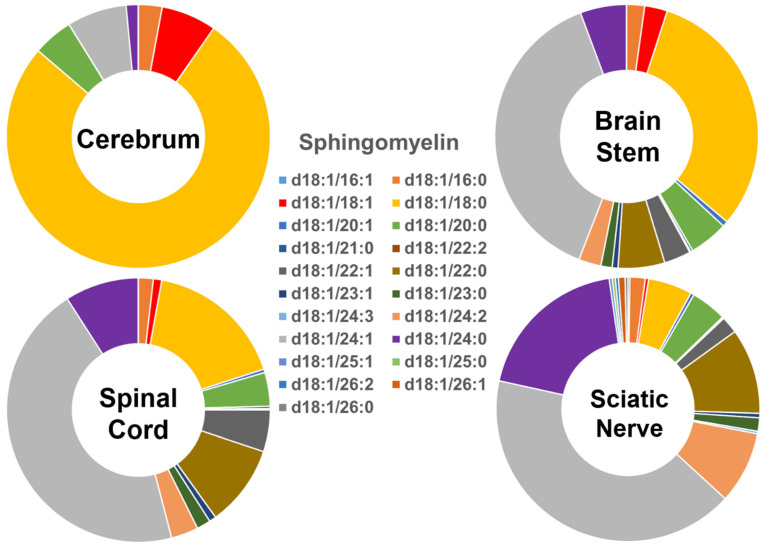
Comparison of sphingomyelin molecular species profiles in cerebrum, brain stem, spinal cord, and sciatic nerve tissues from 2 mo-old mice. Levels of individual sphingomyelin molecular species were assessed via shotgun lipidomics; the relative abundance of each species compared to the total class content is displayed in the form of pie graphs (Excel).

The compositions of the individual SM species found in the four different mouse nervous system tissues were also found to be very distinct (Figure 2), showing a similar pattern to that of the Cer species described above. Specifically, the SM species containing long-chain fatty acids were predominant (>90%) in the cerebrum, while the abundance of those containing very-long-chain fatty acids was very low (<10%). The relative abundance of these two groups of SM species was approximately 40% and 60% in the brain stem, <30% and >70% in the spinal cord, and <15% and >85% in the sciatic nerve, respectively. The SM(d18:1/18:0) species was dominant in the cerebrum, although its abundance decreased gradually from the cerebrum to brain stem, spinal cord, and sciatic nerve. In contrast to the composition of the SM(d18:1/18:0) species, the levels of the SM(d18:1/24:1)/SM(d18:1/24:0) species gradually increased from the cerebrum to the brain stem, spinal cord, and sciatic nerve. The absolute levels of each SM species detected (expressed as nmol/mg of the total protein content) are listed in Supplementary Table S3.

**Figure 3:**
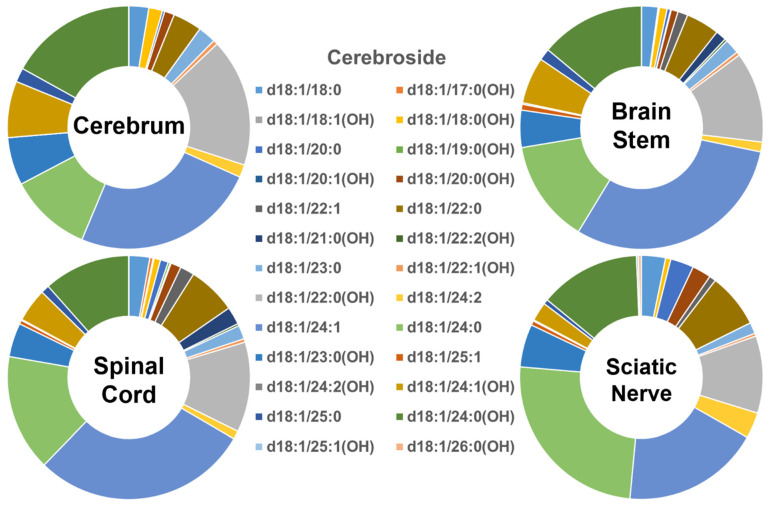
Comparison of cerebroside molecular species profiles in cerebrum, brain stem, spinal cord, and sciatic nerve tissues from 2 mo-old mice. Levels of individual cerebroside molecular species were assessed via shotgun lipidomics; the relative abundance of each species compared to the total class content is displayed in the form of pie graphs (Excel).

In contrast, the profiles of the individual cerebroside species present in the four different mouse nerve tissues (Figure 3) were very different from those of Cer and SM described above. The abundance of the group of cerebroside species containing long-chain fatty amides present in all of the four examined nervous system tissues was low, accounting for only <10% of the total. The majority of cerebroside species were those containing very-long-chain fatty acids, accounting for >90% of the total. The patterns of the individual cerebroside molecular species in the tissues of the cerebrum, brain stem, spinal cord, and sciatic nerve were very similar to each other. Another difference between the cerebroside pattern and those of Cer and SM was the presence of a large amount of the cerebroside species containing a hydroxyl group; this comprised more than 50% of the cerebroside species contained in the hydroxy group in the cerebrum. The content of the hydroxy-containing cerebroside species decreased gradually from the cerebrum to the brain stem, spinal cord, and sciatic nerve (i.e., 52%, 41%, 37%, and 36%, respectively). The absolute levels of each of the cerebroside species detected (expressed as nmol/mg of the total protein content) are listed in Supplementary Table S4.

**Figure 4:**
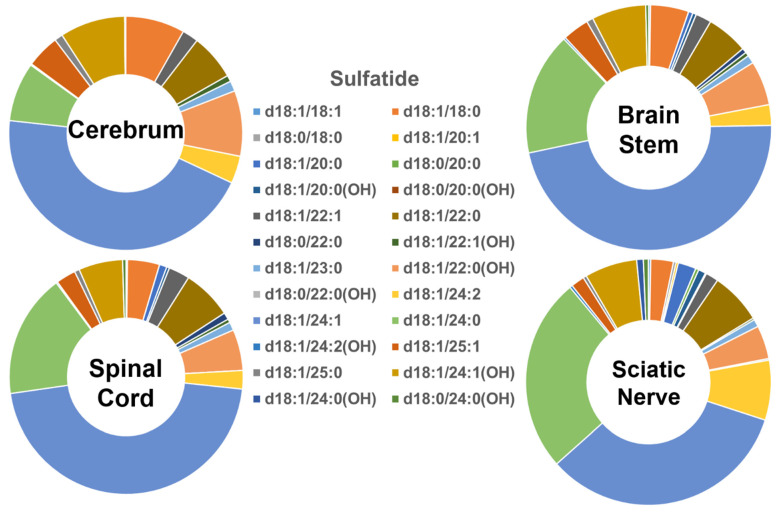
Comparison of sulfatide molecular species profiles in cerebrum, brain stem, spinal cord, and sciatic nerve tissues from 2 mo-old mice. Levels of individual sulfatide molecular species were assessed via shotgun lipidomics; the relative abundance of each species compared to the total class content is displayed in the form of pie graphs (Excel).

**Figure 5:**
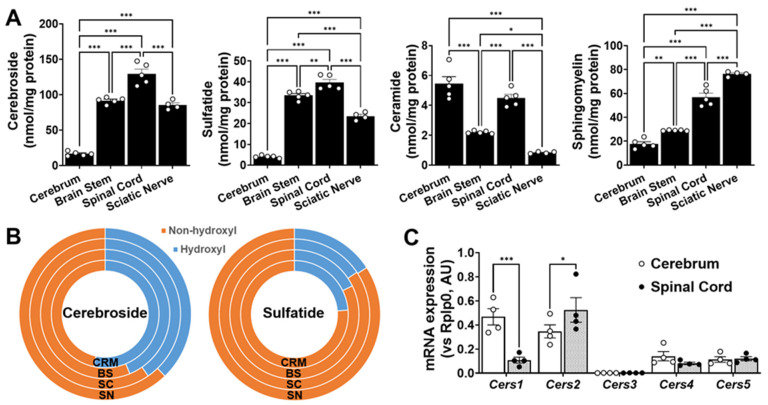
Nervous tissues analyzed display different sphingolipid mass and sphingolipid biosynthesis-related gene expression levels. Total amounts of Cer, SM, cerebroside, and sulfatide in cerebrum (CRM), brain stem (BS), spinal cord (SC), and sciatic nerve (SN). Total mass levels of individual lipid classes were compared between nervous tissues using ordinary one-way (non-paired) ANOVAs followed by Tukey’s multiple comparisons tests (**A**). Comparison of hydroxyl vs. non-hydroxyl molecular species for cerebroside and sulfatide (**B**). Gene expression levels of each ceramide synthase in cerebrum and spinal cord (**C**). Gene expression levels were compared between nervous tissues using two-way (non-paired) ANOVAs, followed by Tukey’s multiple comparisons tests (GraphPad Prism 9 software). Adjusted *p*-value < 0.05 (*), < 0.01 (**), and < 0.001 (***) for all statistical comparisons.

The molecular profiles of the individual sulfatide species in the four different mouse nervous system tissues are shown in Figure 4. The groups containing long- and very-long-chain fatty amides in the sulfatide class in the tissues were comparable to those of cerebroside species (i.e., <10% and >90%, respectively). Similar to the cerebroside class, the patterns of the individual sulfatide molecular species in the tissues of the cerebrum, brain stem, spinal cord, and sciatic nerve were all very similar to each other. However, the content of those sulfatide species containing the hydroxy group differed from the pattern of the cerebroside molecular species (Figure 5B). Specifically, the hydroxy-containing species were less abundant in the sulfatide class compared to the cerebroside class, making up only 23%, 18%, 16%, and 15% of the total sulfatide content in the cerebrum, brain stem, spinal cord, and sciatic nerve, respectively. Another difference between the sulfatide class and cerebroside class was that (3′-sulfo)Galβ-Cer(d18:1/24:1) was the only predominant species in the cerebroside class in all the examined nerve tissues, whereas a few other molecular species were also abundant in the cerebroside class (e.g., HexCer(d18:1/24:0), HexCer(d18:1/24:0(OH)), and HexCer(d18:1/22:0(OH))). The absolute levels of each sulfatide species detected (expressed as nmol/mg of the total protein content) are listed in Supplementary Table S5.

The total absolute levels of the four sphingolipid classes analyzed in the cerebrum, brain stem, spinal cord, and sciatic nerve are summarized in Figure 5, whereas the profiles of the individual molecular species of each of the lipid classes are summarized in Figure S1. In summary, the following points could be drawn from the study: (1) the total content of these sphingolipids increased from the cerebrum to the brain stem, sciatic nerve, and spinal cord, which largely reflects the differences in the myelin content; (2) the sulfatide content was roughly a third of the cerebroside content in all of the tissues, as has been previously reported [12,21]; and (3) the ratios of cerebroside and SM provide some rough information about the abundance of the myelin sheath relative to the neural cell body membrane, since the former is largely present in the myelin, whereas the latter is largely present in the cellular plasma membrane.

In order to better understand the patterns of the sphingolipids in the examined nerve tissues, the mRNA expression levels of the genes that are directly involved in sphingolipid metabolism, including those coding for enzymes involved in *de novo* ceramide synthesis and sphingomyelin hydrolysis, were also measured using RT-PCR (Figure 5C). Cer *de novo* synthesis starts with the condensation of serine and palmitoyl CoA, a reaction that is catalyzed by serine palmitoyltransferase (SPT). No significant differences were found in the expression of Sptlc1 or Sptlc2 between the two extreme tissue types, i.e., the cerebrum and spinal cord (Figure 5C). The acylation of the free primary amine group of sphingoid bases is catalyzed by Cer synthases (CerSs). The levels of the individual CerSs were in the order of CerS1 > CerS2 > CerS4 > CerS5 > CerS3 in the mouse cerebrum and of CerS2 > CerS5 > CerS1 > CerS4 > CerS3 in the mouse spinal cord. The pattern of the CerS expression in the mouse cerebrum was similar to that previously reported in the mouse brain [22]. Our results are also in agreement with the fact that CerS1 catalyzes the synthesis of Cer (d18:1/18:0) and is primarily expressed by the neuronal cells [23,24], while CerS2 catalyzes the synthesis of very-long-acyl-chain ceramides and is primarily expressed by the myelinating cells (i.e., oligodendrocytes in the CNS and Schwann cells in the PNS) [23,24]. Finally, Cer can also be generated via sphingomyelin hydrolysis, a reaction that is catalyzed by a number of sphingomyelinases. Interestingly, the gene expression levels of *Smpd3* were significantly higher in the cerebrum tissue compared to the spinal cord, suggesting that neutral sphingomyelinase activity is higher in the gray matter compared to the white matter. These results are in agreement with the much higher levels of sphingomyelin we observed in the spinal tissue vs. cerebrum, as well as with the high levels of ceramide we observed in the cerebrum tissue, despite the low levels of the more complex sphingolipids such as SM, cerebroside, and sulfatide. On the other hand, the expression levels of *Smpd1* were not significantly altered between both CNS regions, suggesting that the acid sphingomyelinase activity may not be dramatically different between the CNS regions (Figure 5C).

## 3. Discussion

Lipidomics analysis has been applied to a variety of biomedical and biological studies, and has provided useful information and deep insights into the underpinning molecular mechanism(s) that lead to changes in the cellular lipidomes under physiological and pathological conditions [25]. In the current study, we exploited the MDMS-SL technology to reveal a “lipidomic atlas” of selected sphingolipid classes in different regions of the nervous system, including the cerebrum, brain stem, spinal cord, and sciatic nerve. The study reveals that sphingolipid profiles vary drastically within the different nervous system regions in a spatially specific manner. In addition, these distinct sphingolipid profiles are in agreement with the observed region-specific expression levels of the sphingolipid-related genes.

Historically, the acyl-CoA-dependent CerS reaction was first described by Sribney in the 1960s [26]. The studies by Morell et al. [27,28] further suggested that the acyl-CoA-dependent Cer synthesis activity in the mouse brain was governed by more than one enzyme. The regulation of the synthesis of the Cer species containing particular N-acyl-chain lengths by individual CerS (i.e., the acyl-CoA preferences) was characterized by several studies [23,24]. It is well appreciated that CerSs (CerS1 to CerS6) regulate the synthesis of Cer species containing particular acyl-chain lengths and the distribution of the CerSs in mouse brain tissues, based on the data reported by Laviad et al. [22]. In summary, it was found that each CerS isoform displays N-acyl-chain-length specificities as follows: CerS1 mainly generates Cer species containing C18 fatty acyl chains; CerS2 catalyzes the synthesis of Cer species containing C22, C24, and C26 carbon-numbered acyl chains; CerS3 determines the synthesis of Cer species containing C18:0 and C24:0 fatty acyl chains; CerS4 selectively controls the synthesis of Cer species containing C18:0 and C20:0 aliphatic chains; and CerS5 and CerS6 predominantly contribute to the levels of Cer species containing C14:0 and C16:0 fatty acyl chains.

Our data on CerS expression levels in the cerebrum demonstrate that CerS1 and CerS2 were the dominant isoforms. As mentioned above, CerS1 mainly responds to the synthesis of the C18-containing Cer species and CerS2 mainly responds to the synthesis of those containing C22 and C24 aliphatic chains. This is consistent with our results from the lipidomics analysis, i.e., the Cer species containing C18 and C24 length acyl chains were the predominant species in the mouse cerebrum. Moreover, the mRNA profiling of CerS in the spinal cord demonstrated that the CerS2 enzyme was the predominant isoform of CerSs, which was also consistent with the observation that Cer species containing C22 and C24 aliphatic chains were the predominant species in mouse spinal cord.

In addition, the profiles of the determined sphingolipids could also be informative about the role of the individual pathways in different mouse nervous tissues. For example, our lipidomics analysis demonstrated that the profiles of the Cer and SM species were very similar in all of the examined nervous system tissues. This strongly suggests that the enzymes responsible for the synthesis of SM species (i.e., SM synthase 1 and 2) might not have special selectivity in the nervous system. In contrast, the profiles of the cerebroside and sulfatide species were very different from those of the Cer and SM species in the cerebrum, where the N18-containing Cer and SM species were dominant, but the N18-containing cerebroside and sulfatide species were only minor components. This is likely due to the Cer species containing very long fatty acyl chains being more favorable for the synthesis of the cerebroside and sulfatide species within the myelinating cells, as was previously demonstrated [29]. That could lead to the shorter fatty acyl chains of the Cer species for the synthesis of the SM species, as was previously suggested by results obtained in the rat brain [30]. In fact, the profiles of the cerebroside and sulfatide species were similar to each other in all of the examined nerve tissues, whereas the profiles of the Cer and SM species were largely different across these nerve tissues.

The cell-type composition within the different nerve tissues is an important factor to consider as well. Specifically, of the nervous tissues analyzed, the cerebrum had the highest abundance of neurons and the lowest myelin content, which resulted in lower total levels of sphingolipids compared to those in the brain stem, spinal cord, and sciatic nerve—except for Cer, which might be preferably located in neuron cells (particularly Cer N18:0). Therefore, the different levels of cerebroside, sulfatide, SM, and Cer in these four different nervous system tissues provide a good indication of the different neural cell types in these tissues. For example, the ratio of SM vs. cerebroside indicates the constituents of the tissues since SM is enriched in the cell membrane, while cerebroside is virtually exclusively located in the myelin. Thus, the lower the ratio, the fewer the neural cell bodies and the richer the myelin component. It was clear that both the cerebrum and sciatic nerve contain lower myelin than both the brain stem and spinal cord.

In conclusion, the MDMS-SL platform employed in this study enabled us to determine the profiles of four classes of sphingolipids in the mouse cerebrum, brain stem, spinal cord, and sciatic nerve. To understand the profiles of the nervous tissue lipidomes, the mRNA expression of Cer synthases was also analyzed in both the cerebrum and spinal cord. Our results clearly confirm the high selectivity of the different CerSs in the synthesis of specific N-acyl-chain Cer species in the different mouse nerve tissues. To the best of our knowledge, the work presented herein represents the first study to profile the species of the four sphingolipid classes in both the CNS and PNS. These data provide information about the different sphingolipids species, synthesis pathways, and cell types in these nerve tissues to a certain extent. We believe that this type of knowledge should be very useful for understanding the structure and functioning of the individual nervous tissues under pathophysiological conditions.

## 4. Materials and Methods

### 4.1. Materials

The internal standard N-pentadecanoyl cerebroside (HexCer(d18:1/15:0)) was obtained from Matreya LLC, Inc. (Pleasant Gap, PA, USA). The internal standard N-palmitoyl 3′-sulfogalactosylceramide ((3′-sulfo)Galβ-Cer(d18:1/16:0)) was purchased from Cayman Chemical, Inc. (Michigan, MI, USA). Internal standards N-heptadecanoyl-D-erythro-sphingosine (d18:1-N17:0 Cer) and N-lauroyl-D-erythro-sphingosylphosphorylcholine (d18:1-N12:0 SM) were purchased from Avanti Polar Lipids, Inc. (Alabaster, AL, USA). All solvents used for lipid extraction, sample preparation and analysis (including chloroform, methanol, and isopropanol), and a Pierce™ Bicinchoninic Acid (BCA) Protein Assay Kit were obtained from Thermo Fisher Scientific (Pittsburgh, PA, USA). Lithium chloride and lithium hydroxide were purchased from Millipore Sigma Co. (St. Louis, MO, USA). Sample homogenization was performed with Precellys^®^24 Homogenizer and homogenizing ceramic beads kit (2 mL) from Bertin Instruments (Rockville, MD, USA). N-EVAP nitrogen evaporator (24 positions) was obtained from Organomation Associates, Inc. (Berlin, MA, USA).

### 4.2. Animals

Young wild-type C57BL/6 male mice (2–4 mo of age) (5 animals per group) were purchased from the Jackson Laboratory (Bar Harbor, ME). They were fed with a standard rodent chow diet and water ad libitum, and maintained in a temperature- (20–22 °C) and lighting-controlled facility (12 h of light and dark cycle). Animals were euthanized by asphyxiation with CO_2_ followed by decapitation. Different nerve tissue samples including cerebrum, brain stem, spinal cord, and sciatic nerve tissue were dissected, harvested, and immersed into liquid nitrogen immediately. Frozen tissues were stored at −80 °C until lipid extraction and analysis. All animal procedures conducted in the study were in accordance with the “Guide for the Care and Use of Laboratory Animals” (National Research Council of National Academies) and were approved by the UT Health SA Institutional Animal Care and Use Committee (IACUC).

### 4.3. Sample Preparation

Mouse cerebrum, brain stem, spinal cord, and sciatic nerve tissue samples were homogenized in 10-times diluted PBS using a Precellys Evolution^®^ Homogenizer at 6000× *g* rpm for 20 s and paused for 10 s at 4 °C for 3 consecutive cycles. The protein content of each individual homogenate was determined via Pierce BCA assay kit. The rest of the homogenate was transferred into a disposable glass culture test tube for lipid extraction. For quantification of lipid species, four lipid internal standards were added prior to lipid extraction. Lipid extraction was performed with a modified procedure of Bligh and Dyer extraction as previously described [31,32]. Briefly, a certain amount of four internal standard mixture was added into each sample prior to lipid extraction based on the protein concentration of each individual sample. Lipid levels were normalized to total protein content. Twelve ml of chloroform/methanol/50 mM lithium chloride water solution (*v*/*v*/*v*, 1/1/1) were added into individual samples and vortexed for 1 min. Then, all of the samples were centrifuged at 4000× *g* for 10 min at room temperature. The chloroform layer of each individual sample was collected into a new glass tube. This procedure was repeated once by adding 4 mL of chloroform and the chloroform layer was combined with the first extract. The extract solvent was evaporated under a nitrogen stream. The lipid residues were re-extracted using the aforementioned protocol with 12 mL of chloroform/methanol/10 mM lithium chloride water solution (*v*/*v*/*v*, 1/1/1). The extract solvent was evaporated under a nitrogen stream. Finally, each lipid extract was resuspended with a volume of 500 μL of chloroform/methanol (1:1, *v*/*v*) per mg of original tissue protein. Individual lipid solutions were flushed with nitrogen, capped, and stored at −20 °C for lipid analysis. Individual lipid extracts were further diluted with chloroform/methanol/isopropanol (1/2/4, *v*/*v*/*v*) and with or without a small amount of lithium hydroxide in methanol prior to direct infusion for MS analysis. The lithium hydroxide solution was made of a 4000 times dilution of a saturated methanol solution of lithium hydroxide.

### 4.4. Mass Spectrometric Analysis

Mass spectrometry (MS) and tandem MS analyses of lipids were performed with a QqQ mass spectrometer (TSQ Quantiva, Thermo Fisher Scientific, San Jose, CA, USA) and a linear ion trap mass spectrometer (Thermo LTQ Orbitrap XL, San Jose, CA, USA), both equipped with an automated nanospray device (Advion Bioscience, Ithaca, NY, USA) as previously described [33,34]. Prior to direct infusion, individual lipid extract solutions were diluted in CHCl_3_/MeOH/isopropyl alcohol (1:2:4, *v*/*v*/*v*) to an estimated concentration of ~100 pmol/μL to avoid possible lipid aggregation. Tandem MS analysis of a diluted lipid solution was conducted under fixed collision gas pressure of 1.0 mTorr, while the collision energy was varied to achieve optimized conditions, as described previously [33].

### 4.5. PCR Analysis of Ceramide Synthase Expression

Total RNA was isolated after homogenizing nerve tissues in Invitrogen™ TRIzol™ reagent (Thermo Scientific) using a Direct-zol™ RNA MiniPrep kit for RNA isolation (Zymo Research, Catalog #R2050) as previously described [12]. RNA concentrations were quantified using a NanoDrop 8000 spectrophotometer (Thermo Scientific). mRNA was reversely transcribed from total RNA using an Applied Biosystems™ High-Capacity cDNA Reverse Transcription Kit (Thermo Scientific, Catalog #4368814). qRT-PCR was performed using SsoFast™ EvaGreen^®^ Supermix (Bio-Rad) and a two-step program (60 °C annealing temperature) on a LightCycler^®^ 480 Instrument II (Roche). The expression of Rplp0 (a.k.a., 36B4), a commonly used universal reference gene that codes for the acidic ribosomal phosphoprotein P0, was used to normalize relative expression levels of sphingolipid metabolism-related genes. The primer sequences used are provided in Supplementary Table S1.

### 4.6. Data Analysis

All mass spectral data were automatically acquired by a customized sequence subroutine operated under Xcalibur software [34]. Data processing was conducted as previously described [33] based on the principles of shotgun lipidomics such as selective ionization, low concentration of lipid solution, and correction for differential kinetics of fragmentation [35,36]. All data were obtained from five separate animals. Total mass levels of individual lipid classes were compared between nervous tissues using ordinary one-way (non-paired) ANOVAs followed by Tukey’s multiple comparisons tests. Gene expression levels of different nervous tissues were compared using two-way (non-paired) ANOVAs followed by Tukey’s multiple comparisons tests (GraphPad Prism software). Significance was set at the levels of adjusted *p*-values < 0.05 (*), < 0.01 (**), and < 0.001 (***) for all statistical comparisons. Lipidomics data, expressed as nmol of a given lipid class or molecular species per mg of total protein content, were either displayed as dot plots (data represent the mean ± SEM) for total class contents or as multiple-ring “pie/doughnut” graphs (Microsoft Excel) to display the relative abundance of individual molecular species or species clusters within a given class.

## Supplementary Materials

The following are available online at https://www.mdpi.com/article/10.3390/ijms222111358/s1.
