# Supplementary material for: A Lipidomics Atlas of Selected Sphingolipids in Multiple Mouse Nervous System Regions
Source: Int J Mol Sci. 2021 Oct 21;22(21):11358. doi: 10.3390/ijms222111358 (PMC8583963; doi:10.3390/ijms222111358)
Supplement: Supplementary file 1 [file ijms-22-11358-s001.zip › ijms-1416794-supplementary.pdf]

International Journal of *Molecular Sciences*

MDPI

Supplementary Materials

A Lipidomics Atlas of Selected Sphingolipids in Multiple Mouse Nervous System Regions

Chunyan Wang [1,†], Juan Pablo Palavicini [1,2] and Xianlin Han [1,2,*]

1 Barshop Institute for Longevity and Aging Studies, University of Texas Health Science Center at San Antonio, 4939 Charles Katz Drive, San Antonio, TX 78229, USA; chunyanw123@gmail.com (C.W.); PalaviciniJ@uthscsa.edu (J.P.P.)
2 Department of Medicine, Division of Diabetes, University of Texas Health Science Center at San Antonio, San Antonio, TX 78229, USA
* Correspondence: hanx@uthscsa.edu; Tel.: +1-(210)-562-4104
† Current address: Center for Human Nutrition, Washington University School of Medicine, 660 S. Euclid Ave., St. Louis, MO 63130 USA.

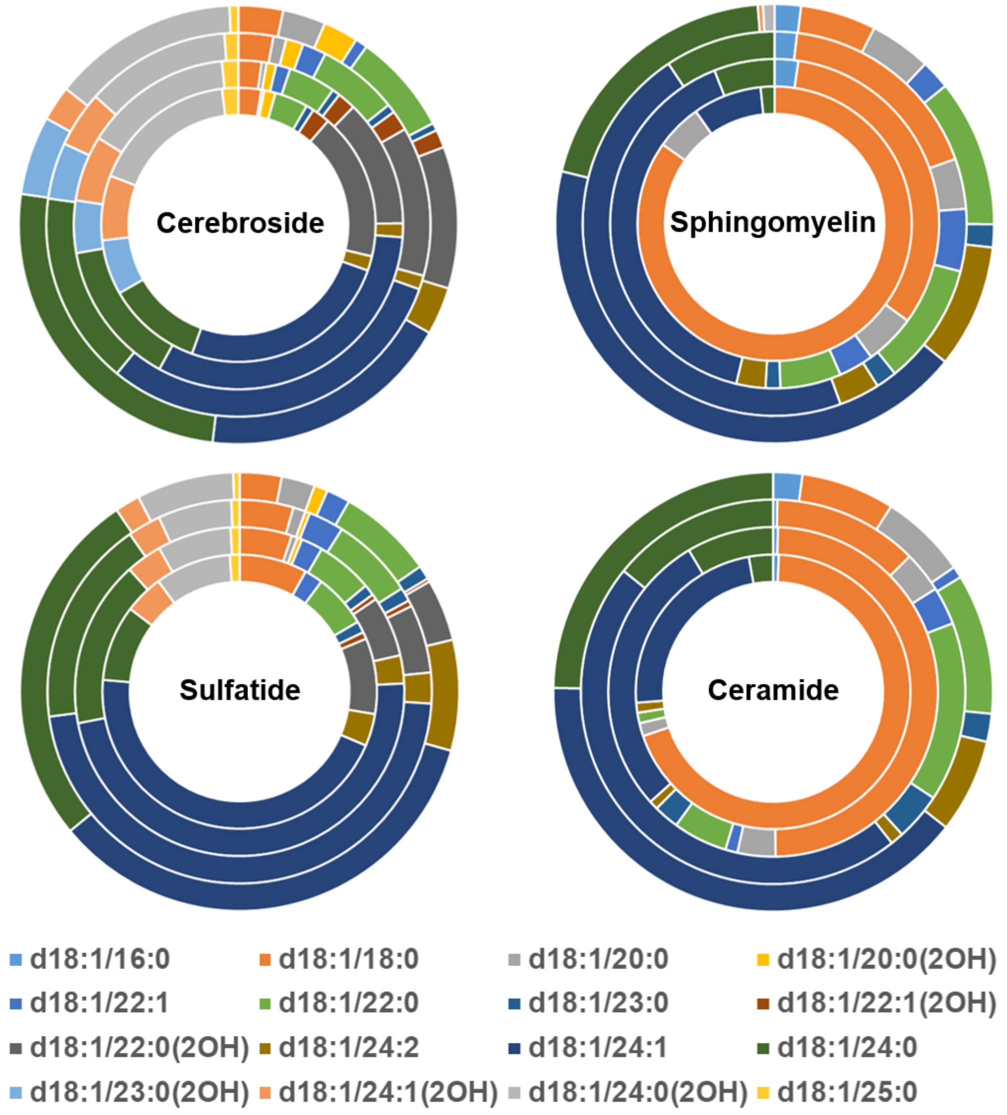

Figure S1. Summary of sphingolipid profiles in cerebrum, brain stem, spinal cord, and sciatic nerve tissues from 2 mo-old mice (from inner to outer-most ring, respectively). Each molecular species is represented by a different color.

Table S1. Primer sequences used for gene expression studies in mouse nervous system tissues.

Name	Sequence (5′ → 3′)
Cers1 Frwd	CGTAAGGACTCGGTGGTCAT
Cers1 Rev	GCGTAGGAAGAGGCAATGAG
Cers2 Frwd	GTTTAACTACGCGGGATGGA
Cers2 Rev	GGCGAACACAATGAAGAGGT
Cers3 Frwd	GCTACACCTCTAGCAAATGCAC
Cers3 Rev	ATCTTTCAACCTGGCGCTCT
Cers4 Frwd	GATGAAGCCTCTCTGCTGCT
Cers4 Rev	AGGACACCCACAGGTTTCTG
Cers5 Frwd	CATGCCATCTGGTCCTACCT
Cers5 Rev	CATCACTGCGGTCATCCTTA
Sptlc1 Frwd	TCGAGTTAAGGCCACAGCTT
Sptlc1 Rev	CATAGAACCCTCGAGGACCA
Sptlc2 Frwd	GGATACATCGGAGGCAAGAA
Sptlc2 Rev	GACATCGACGTGGCATACAC
Smpd1 Frwd	CAGTTCTTTGGCCACACTCA
Smpd1 Rev	CGGCTCAGAGTTTCCTCATC
Smpd3 Frwd	CTGACTCCAGACAGCATCCA
Smpd3 Rev	ACTGTGCTGAGCTTGGGACT
36B4 Frwd	TGGAAGTCCAACTACTTCCTCAA
36B4 Rev	ATCTGCTGCATCTGCTTGGAG

Table S2. Ceramide species and their mass levels (nmol/mg protein) in the samples of cerebrum, brain stem, spinal cord, and sciatic nerve tissue..

Cer	Cerebrum	Brain Stem	Spinal cord	Sciatic Nerve
Cer (d18:1/16:0)	0.04±0.00	0.01±0.01	0.02±0.01	0.02±0.01
Cer (d18:1/18:0)	3.77±0.69	1.08±0.07	0.55±0.28	0.06±0.00
Cer (d18:1/20:0)	0.08±0.00	0.08±0.01	0.15±0.03	0.05±0.01
Cer (d18:1/22:1)		0.03±0.01	0.14±0.02	0.01±0.00
Cer (d18:1/22:0)	0.07±0.01	0.12±0.02	0.68±0.12	0.09±0.01
Cer (d18:1/23:0)		0.05±0.01	0.18±0.03	0.02±0.01
Cer (d18:1/24:2)	0.06±0.01	0.02±0.01	0.05±0.01	0.06±0.01
Cer (d18:1/24:1)	1.28±0.19	0.62±0.04	2.08±0.29	0.33±0.02
Cer (d18:1/24:0)	0.16±0.04	0.19±0.02	0.64±0.09	0.21±0.02
Sum	5.46±0.92	2.20±0.09	4.49±0.49	0.84±0.06

Table S3. Sphingomyelin species and their mass levels (nmol/mg protein) in cerebrum, brain stem, spinal cord, and sciatic nerve tissue.

SM	Cerebrum	Brain Stem	Spinal cord	Sciatic Nerve
SM(d18:1/16:1)				0.19±0.01
SM(d18:1/16:0)	0.26±0.05	0.32±0.01	0.52±0.09	1.43±0.04
SM(d18:1/18:1)	0.60±0.11	0.40±0.03	0.29±0.06	0.31±0.03
SM(d18:1/18:0)	6.84±1.31	4.53±0.15	4.89±0.55	4.11±0.09
SM(d18:1/20:1)		0.10±0.02	0.12±0.02	0.33±0.06
SM(d18:1/20:0)	0.44±0.11	0.69±0.02	1.17±0.17	3.35±0.11
SM(d18:1/21:0)		0.04±0.01	0.08±0.03	0.09±0.04
SM(d18:1/22:2)		0.02±0.01	0.05±0.01	0.07±0.01
SM(d18:1/22:1)		0.48±0.04	1.45±0.17	1.60±0.09
SM(d18:1/22:0)		0.83±0.06	2.83±0.35	8.00±0.37
SM(d18:1/23:1)		0.10±0.02	0.24±0.03	0.41±0.04
SM(d18:1/23:0)		0.20±0.02	0.49±0.04	1.25±0.08
SM(d18:1/24:3)				0.23±0.03
SM(d18:1/24:2)		0.40±0.02	0.95±0.10	6.72±0.34
SM(d18:1/24:1)	0.65±0.13	5.58±0.05	12.80±1.50	31.76±0.61
SM(d18:1/24:0)	0.13±0.07	0.83±0.05	2.58±0.37	14.71±0.75
SM(d18:1/25:1)				0.29±0.03
SM(d18:1/25:0)				0.28±0.02
SM(d18:1/26:2)				0.28±0.04
SM(d18:1/26:1)				0.63±0.03
SM(d18:1/26:0)				0.26±0.05
Sum	8.93±1.74	14.52±0.16	28.46±3.36	76.30±1.71

Table S4. Cerebroside species and their mass levels (nmol/mg protein) in cerebrum, brain stem, spinal cord, and sciatic nerve tisue.

HexCer	Cerebrum	Brain Stem	Spinal cord	Sciatic Nerve
HexCer(d18:1/18:0)	0.44±0.09	0.27±0.17	3.60±0.34	2.75±0.19
HexCer(d18:1/17:0(OH))			0.56±0.06	
HexCer(d18:1/18:1(OH))			0.16±0.05	
HexCer(d18:1/18:0(OH))	0.30±0.06	0.18±0.12	1.13±0.15	0.59±0.06
HexCer(d18:1/20:0)	0.05±0.02	0.04±0.02	1.35±0.20	2.65±0.31
HexCer(d18:1/19:0(OH))			0.38±0.08	
HexCer(d18:1/20:1(OH))			0.15±0.08	
HexCer(d18:1/20:0(OH))	0.22±0.04	0.13±0.09	1.79±0.17	2.15±0.22
HexCer(d18:1/22:1)			2.45±0.27	0.75±0.08
HexCer(d18:1/22:0)	0.64±0.11	0.38±0.26	8.35±0.93	6.21±0.93
HexCer(d18:1/21:0(OH))			3.06±0.25	
HexCer(d18:1/22:2(OH))			0.38±0.04	
HexCer(d18:1/23:0)/ HexCer(d18:1/22:1(OH))	0.50±0.09	0.29±0.20	2.97±0.36	1.59±0.23
HexCer(d18:1/22:0(OH))	2.83±0.45	1.64±1.19	15.46±1.51	8.75±0.46
HexCer(d18:1/24:2)	0.28±0.04	0.16±0.12	1.44±0.26	2.94±0.54
HexCer(d18:1/24:1)	4.09±0.63	2.36±1.73	37.38±3.96	15.64±1.25
HexCer(d18:1/24:0)	1.82±0.36	1.09±0.73	20.01±2.10	21.24±2.54
HexCer(d18:1/23:0(OH))	1.06±0.16	0.61±0.45	5.83±0.67	4.86±0.47
HexCer(d18:1/25:1)/ HexCer(d18:1/24:2(OH))			0.83±0.13	0.63±0.25
HexCer(d18:1/24:1(OH))/ HexCer(d18:1/25:0)	1.56±0.23	0.90±0.66	7.21±0.67	2.70±0.37
HexCer(d18:1/24:0(OH))	2.81±0.49	1.65±1.16	14.95±1.56	11.48±0.77
HexCer(d18:1/25:1(OH))/ HexCer(d18:1/25:0)				0.22±0.05
HexCer(d18:1/26:0(OH))				0.32±0.11
Sum	16.59±2.69	9.64±6.95	129.47±13.42	85.47±5.50

Table S5. Sulfatide species and their mass levels (nmol/mg protein) in cerebrum, brain stem, spinal cord, and sciatic nerve tissue.

Sulfatides	Cerebrum	Brain Stem	Spinal cord	Sciatic Nerve
(3'-sulfo)Galβ-Cer(d18:1/18:1)		0.08±0.00	0.07±0.01	0.07±0.00
(3'-sulfo)Galβ-Cer(d18:1/18:0)	0.31±0.12	1.69±0.18	1.76±0.11	0.70±0.05
(3'-sulfo)Galβ-Cer(d18:0/18:0)				0.08±0.01
(3'-sulfo)Galβ-Cer(d18:1/20:1)				0.07±0.01
(3'-sulfo)Galβ-Cer(d18:1/20:0)		0.19±0.03	0.40±0.04	0.56±0.05
(3'-sulfo)Galβ-Cer(d18:0/20:0)				0.10±0.01
(3'-sulfo)Galβ-Cer(d18:1/20:0(OH))		0.16±0.04	0.15±0.13	0.23±0.02
(3'-sulfo)Galβ-Cer(d18:0/20:0(OH))				0.03±0.01
(3'-sulfo)Galβ-Cer(d18:1/22:1)	0.08±0.03	0.70±0.03	1.16±0.13	0.40±0.03
(3'-sulfo)Galβ-Cer(d18:1/22:0)	0.24±0.06	1.83±0.16	2.74±0.14	1.60±0.11
(3'-sulfo)Galβ-Cer(d18:0/22:0)		0.21±0.04	0.38±0.05	
(3'-sulfo)Galβ-Cer(d18:1/22:1(OH))	0.03±0.02	0.18±0.02	0.20±0.02	0.06±0.01
(3'-sulfo)Galβ-Cer(d18:1/23:0)	0.05±0.02	0.36±0.03	0.45±0.04	0.23±0.02
(3'-sulfo)Galβ-Cer(d18:1/22:0(OH))	0.34±0.09	1.96±0.36	2.24±0.31	1.03±0.06
(3'-sulfo)Galβ-Cer(d18:0/22:0(OH))				0.05±0.00
(3'-sulfo)Galβ-Cer(d18:1/24:2)	0.15±0.03	0.91±0.06	1.01±0.17	1.85±0.16
(3'-sulfo)Galβ-Cer(d18:1/24:1)	1.73±0.31	15.71±0.53	18.26±2.04	7.87±0.51
(3'-sulfo)Galβ-Cer(d18:1/24:0)	0.34±0.19	5.41±0.31	6.78±0.50	6.01±0.41
(3'-sulfo)Galβ-Cer(d18:1/24:2(OH))	0.01±0.00	0.09±0.01	0.08±0.01	0.08±0.01
(3'-sulfo)Galβ-Cer(d18:1/25:1)	0.01±0.01			
(3'-sulfo)Galβ-Cer(d18:1/25:0)/ (3'-sulfo)Galβ-Cer(d18:1/24:1(OH))	0.22±0.04	1.46±0.13	1.35±0.12	0.52±0.04
(3'-sulfo)Galβ-Cer(d18:1/24:0(OH))	0.34±0.09	2.39±0.32	2.41±0.31	1.63±0.10
(3'-sulfo)Galβ-Cer(d18:0/24:0(OH))				0.21±0.01
(3'-sulfo)Galβ-Cer(d18:1/26:1)	0.01±0.01	0.13±0.02	0.19±0.02	0.16±0.01
Sum	3.88±0.74	33.45±1.78	39.66±3.02	23.96±1.58
